# Development and validation of an AI-based application for early detection and risk stratification of oral potentially malignant disorders

**DOI:** 10.1016/j.jobcr.2025.10.017

**Published:** 2025-10-28

**Authors:** Akash Gajanan Prabhune, Vinay R. Srihari, Shreya Shree, Manish Katiyar, Vipin Thampi

**Affiliations:** a ADMIRE Centre for Advancing Digital Health, Institute of Health Management Research Bangalore, India; b Project Research Scientist III, All India Institute of Medical Sciences, Madurai, Tamil Nadu, India

**Keywords:** Oral potentially malignant disorders (OPMDs), Artificial intelligence (AI), Deep learning, Early detection, Oral cancer screening, Community health applications

## Abstract

**Background:**

Oral Potentially Malignant Disorders (OPMDs) are early indicators of oral cancer, and timely detection is essential for improving patient outcomes. However, diagnosis often relies on expert clinical evaluation, which may not be available in low-resource settings.

**Objective:**

This study presents the development and validation of PRAYAAS, an AI-based mobile application for early detection and risk stratification of OPMDs using intraoral images.

**Methods:**

A total of 794 intraoral images were classified into three categories: (1) Normal mucosa/inflammatory conditions, (2) Premalignant conditions, and (3) Oral carcinoma. Images were split into training (70 %), validation (18 %), and test (12 %) datasets while maintaining class balance. Preprocessing involved resizing to 224 × 224 pixels, contrast enhancement, and normalization. A U-Net-based model segmented lesion regions, followed by classification using a fine-tuned DenseNet201 model. Model performance was evaluated using accuracy, precision, recall, F1-score, and confusion matrices.

**Results:**

The DenseNet201 classifier achieved 94 % accuracy on the test set. For normal/inflammatory lesions, precision and recall were 1.00. For premalignant lesions, precision was 0.87 and recall was 1.00. For carcinoma, precision was 1.00 and recall was 0.80. The integrated segmentation module improved lesion focus and reduced background noise. The app provides class-wise risk scores and a user-friendly interface for clinical support.

**Conclusion:**

PRAYAAS offers a robust, mobile-enabled solution for early OPMD screening. By integrating segmentation and classification into a single platform, the tool holds promise for enhancing community-based oral cancer detection and referral.

## Graphical abstract

**Figure:**
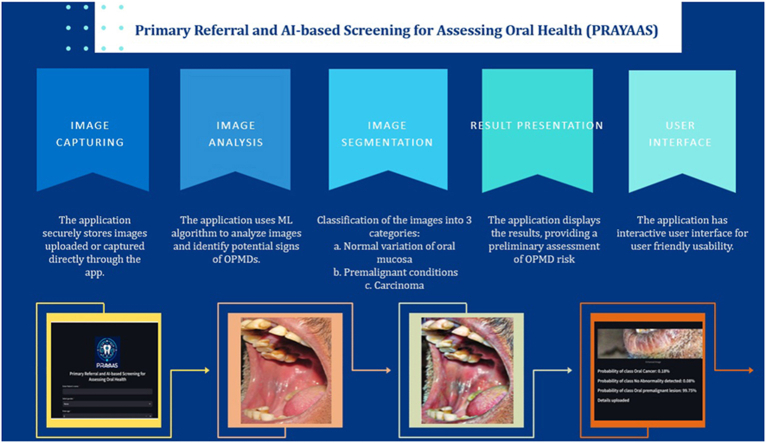

## 1 Background

Oral cancer is a significant global health concern, with a particularly high incidence in South and Southeast Asia, especially in India. In India, oral cancer is the most common cancer among men and the third most common among women. It accounts for 30 % of all cancers in the country.^1^ The age-standardised incidence rate in India is 9.1 per 100,000, with higher rates of 13.9 and 4.3 per 100,000 among men and women, respectively.^2^ These high rates, coupled with late-stage diagnoses, contribute to substantial morbidity and mortality associated with oral cancer.

Oral potentially malignant disorders (OPMDs) are the forerunners of oral cancer. OPMDs are clinically detectable chronic lesions that can progress to cancer. The presence of an OPMD is itself a risk factor for oral cancer.^3^ Early detection and treatment of OPMDs is therefore a vital strategy in controlling oral cancer.^3^,^4^,^5^ The malignant transformation of OPMDs may take from 5 to 10 years, which provides a window for prevention through treatment of OPMDs.^6^ India has a high incidence of OPMDs that progress to oral cancer, making early detection of OPMDs an effective cancer control measure.

Early screening is crucial because many oral cancers are diagnosed at advanced stages, which reduces the effectiveness of treatment. Screening aims to identify those who probably have the disease from those who probably do not, before the onset of symptoms. In the case of oral cancer, screening is designed to detect both oral cancer and OPMDs. Screening can lead to the down-staging of disease and a reduction in mortality and morbidity.^7^ Although some developed countries provide oral screening as part of general health screenings, no national screening programs for oral cancer exist. Most national organisations have not recommended population-based screening due to lack of sufficient evidence that it reduces oral cancer mortality.^8^ The American Cancer Society and the American Dental Association recommend visual oral examinations (VOE) as part of routine checkups.^9^

Implementing large-scale OPMD screening programs is important, especially in countries like India where the burden of oral cancer is high. Opportunistic screening in dental practices is recommended where healthcare resources are high, although there is limited evidence that it leads to downstaging of the disease. In low-resource settings, the feasibility of using primary health care workers (PHCWs) for oral cancer screening has been tested with good outcomes, as demonstrated by studies showing their effectiveness in identifying and referring suspicious lesions during community-based interventions.^10^,^11^ In India, the National Programme for Prevention and Control of Cancer, Diabetes, Cardiovascular Diseases and Stroke (NPCDCS) recommends population-based screening for oral cancer by frontline health workers. However, data from the National Family Health Survey (NFHS-5) shows that screening rates remain very low, with only 0.9 % and 1.2 % of women and men, respectively, aged 30–49 years having ever undergone oral cancer screening^12^. This suggests the strategy is under-utilized.

Technology can play a significant role in enabling early screening at the household or community level. Mobile health (mHealth) approaches using mobile applications, for example, can aid in data collection, image capture, and remote early detection of oral cancer by community health workers.^13^ These electronic methods also help with maintaining medical records and patient monitoring. Community health workers, with training and technological support, can effectively perform oral cancer screenings in resource-constrained settings.

The current study explores the feasibility of development of an easy-to-use Machine Learning algorithm for detection of OPMDs, andtargeted at community health workers.

## 2 Materials and methods

### 2.1 Data sets

The data for this study was acquired in two phases. Phase 1 utilized an anonymized dataset of 134 intraoral images obtained from a tertiary oral pathology centre located in South India. These images were brush-biopsy-confirmed and included cases of normal oral mucosa, inflamed mucosa, oral pre-malignant lesions, and frank carcinoma. Phase 2 involved the collection of 610 anonymized clinical images from community-based oral health screening camps conducted in semi-urban and rural regions across Karnataka and Tamil Nadu. These images were labeled and provisionally diagnosed by oral pathology experts. All data collection procedures were conducted under institutional ethical approval, and all images were anonymized prior to analysis.

**Fig. 1:**
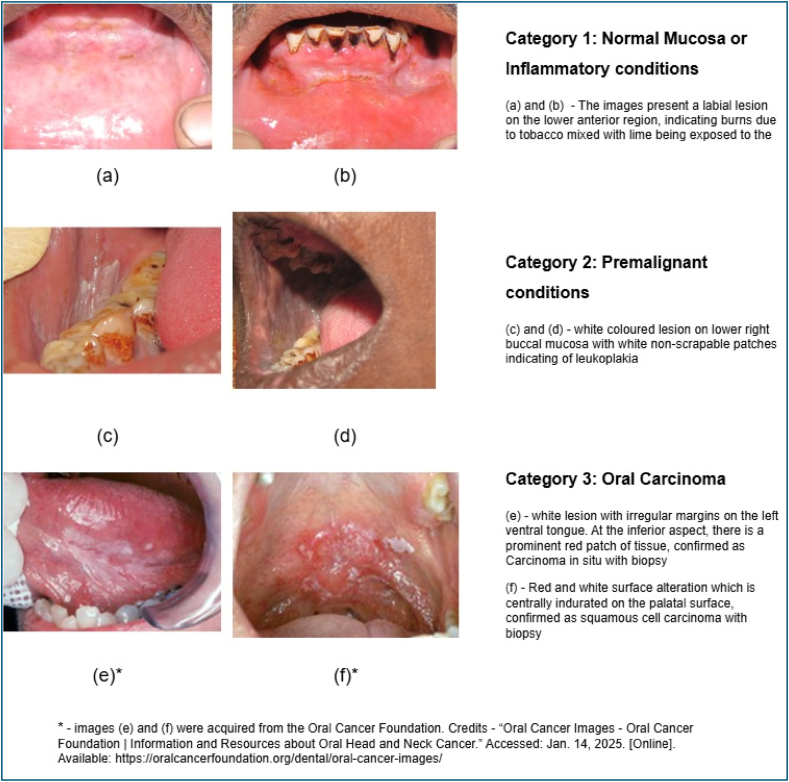
Illustration of three categories of images used for development of algorithm.

The images were labeled into three categories as; Category 1: Normal Mucosa or Inflammatory conditions like burns, tobacco pouch keratosis. Category 2: Premalignant conditions (such as leukoplakia, erythroplakia, or oral submucous fibrosis). Category 3: Oral Carcinoma, the clinical images and their classification is presented in Fig. 1. The dataset used for model development comprised 794 intraoral images. To maintain class balance during model training and evaluation, the dataset was split into 556 images for training (70 %), 142 for validation (18 %), and 96 for testing (12 %). Specifically, the normal/inflammatory class included 190 training, 44 validation, and 12 test images. The premalignant class comprised 210 training, 31 validation, and 31 test images. For carcinoma, 156 images were used for training, 67 for validation, and 41 for testing. This stratified approach ensured that each data subset was representative of the overall class distribution and enabled consistent performance evaluation across categories.

### 2.2 Model development and training

The development of the AI-based application followed the Scrum methodology, an iterative and incremental framework that ensures agility and adaptability in software development. The Scrum process was divided into four sprints, focusing on iterative progress and continuous feedback to enhance model performance and application usability. For model selection, multiple deep learning architectures were evaluated, including VGGNet, ResNet, MobileNetV2, a custom shallower CNN, and DenseNet201. While DenseNet201 initially achieved a validation accuracy of 87 % during the model selection phase, it demonstrated superior performance over lighter alternatives when fully trained and fine-tuned. MobileNetV2 and the custom CNN achieved test accuracies of 88.5 % and 84.2 %, respectively.

The model was fine-tuned using transfer learning with a dataset of intraoral images, classified into three categories: normal mucosa, premalignant lesions, and carcinoma. The training dataset constituted 70 % of the data, while 18 % was reserved for validation and 12 % for testing.

The DenseNet201 model was trained using cross-entropy loss and the Adam optimizer, with a learning rate scheduler to adjust during training. Data preprocessing included resizing all images to 224 × 224 × 3 pixels to match the input size expected by the DenseNet201 architecture, along with contrast enhancement and image normalization. No explicit noise removal filters were applied. Data augmentation techniques simulated real-world scenarios such as variations in lighting and image quality. The DenseNet201 is a deep learning model that enhances gradient flow and reduces computational complexity by connecting each layer to every other layer, promoting efficient feature reuse. With 201 layers, it uses dense blocks to capture rich spatial features and transition layers for down sampling. DenseNet201 excels in image classification tasks, especially in medical imaging, due to its ability to extract detailed patterns and mitigate the vanishing gradient problem. In this study, DenseNet201 was fine-tuned to classify intraoral images into three categories—normal mucosa, premalignant conditions, and carcinoma—demonstrating high accuracy and robust performance.

The algorithm model architecture was built using the DenseNet201 backbone from the Keras applications library with *include_top=False*, which excludes the original classification layers trained on ImageNet. The input images were resized to 224 pixels × 224 pixels × 3 (RGB) before being passed to the model. The DenseNet201 base was initialized with pretrained ImageNet weights and was fully fine-tuned during training (i.e., no layers were frozen) to allow domain-specific feature learning. A custom classification head was added, consisting of the following layers:

• *“GlobalAveragePooling2D″* to reduce the spatial dimensions of the feature map
• *Dense (224, activation='relu’*) to learn non-linear transformations
• *Dropout(0.5)* for regularization
• *Dense(3, activation='softmax’)* as the output layer for predicting the three target classes: normal mucosa/inflammatory, premalignant lesions, and carcinoma.

This architecture allowed effective transfer learning while adapting to the specific patterns observed in intraoral medical images.

A batch size of 32 was selected after pilot experiments with 16, 32 and 64, as it provided the best trade-off between stable gradient updates and available GPU memory. DenseNet201 inherently incorporates Batch Normalization layers after each convolution, and its dense connectivity design mitigates vanishing-gradient issues. These architectural features, together with the Adam optimizer and dynamic learning-rate scheduling, ensured stable convergence without gradient degradation.

The base model, DenseNet201, was initialized with pretrained ImageNet weights (weights = 'imagenet’). We experimented with both partial fine-tuning—freezing early convolutional blocks—and full fine-tuning. Empirical results indicated that full fine-tuning led to higher validation accuracy (87 %) and better generalization across all diagnostic categories. To prevent overfitting, we employed dropout (0.5), early stopping, and learning rate scheduling.

The model was trained with **Adam** *(initial learning rate = 1 × 10*^*−4*^*, β*_*1*_ = *0.9, β*_*2*_ = *0.999, ε = 1 × 10*^*−7*^*, AMSGrad = False)*. A **ReduceLROnPlateau** scheduler *(factor = 0.1, patience = 3, cooldown = 1, min_lr = 1 × 10*^*−6*^*)* adjusted the learning rate based on validation loss, and **EarlyStopping** *(patience = 15, restore_best_weights = True)* curtailed overfitting. We evaluated alternative Adam β settings—(0.95, 0.999) and (0.9, 0.99)—and found the default β_1_/β_2_ = 0.9/0.999 provided the most stable convergence and highest validation performance. A before/after summary of optimizer tuning is provided in Table 1.

**Table 1:** Details of Adam Optimizer settings applied for model training.

Adam settings	Val. accuracy	Macro F1
Before tuning (lr = 1e-3, β1 = 0.9, β2 = 0.999)	79 %	0.72
Tuned (lr = 1e-4, **β1=0.9, β2=0.999**, ε = 1e-7)	**87 %**	**0.82**
Tuned (lr = 1e-4, β1 = 0.95, β2 = 0.999)	85 %	0.80
Tuned (lr = 1e-4, β1 = 0.9, β2 = 0.99)	84 %	0.79

∗We also tested AMSGrad = True and AdamW (wd = 1e-4) but did not observe consistent gains; therefore, we retained Adam with the configuration above.

Hyperparameter optimization was performed using a manual search strategy. Key parameters tuned included initial learning rate (1e-2 to 1e-4), batch size (16–64), dropout rate (0.3–0.7), and optimizer settings. The final configuration—Adam optimizer with initial learning rate 1e-4, batch size 32, and dropout 0.5—yielded an increase in validation accuracy from 79 % to 87 % and macro F1-score from 0.72 to 0.82.

### 2.3 Image Analysis and segmentation

Image analysis and segmentation were critical steps in the workflow to enhance the classification of OPMDs. Preprocessing was applied to standardize image inputs, which included resizing images to 224 × 224 pixels, applying contrast enhancement, and normalizing pixel values. For image segmentation, the focus was on isolating regions of interest, such as lesions or abnormal tissue, from the surrounding oral cavity structures. A U-Net-based architecture was utilized for advanced segmentation, effectively delineating lesion boundaries and separating abnormal regions (e.g., leukoplakia, submucosal fibrosis) from healthy mucosa. This segmentation process ensured that the model concentrated on key features of the lesions during training and validation, reducing misclassification caused by irrelevant background data. The segmented regions were directly input into the classification models, improving diagnostic precision across the OPMD categories.

### 2.4 Output layer and loss configuration

The final classification block consisted of a fully connected Dense layer with three output neurons (logits), corresponding to the diagnostic categories of normal mucosa/inflammatory conditions, premalignant lesions, and carcinoma. We trained the network using categorical cross-entropy loss with the argument from_logits = *True*, which internally applies the SoftMax operation during loss computation. This design follows the recommended practice in TensorFlow/Keras for multi-class classification it preserves numerical stability by avoiding the redundant application of SoftMax both in the model output and inside the loss function, while allowing the logits to be converted to class probabilities via tf.nn.softmax() at inference when probability estimates are required.

### 2.5 Scrum methodology implementation

The Scrum framework structured the development into sprints, each with specific deliverables, Table 2 presents the application of mHealth framework for software development lifecycle processes.

**Table 2:** Agile framework for development of an AI-Based OPMD screening application.

Sprint	Sprint Goal	Activities	Deliverables
**1**	Problem identification and dataset creation	Literature review, dataset collection, and preprocessing pipeline development	Cleaned and pre-processed dataset
**2**	Model training and selection	Evaluation of various AI architectures (VGGNet, ResNet, DenseNet); initial training setup	Selection of DenseNet201 based on accuracy
**3**	Application integration	Integration of the trained model into the application; UI/UX design	Functional alpha version of the mobile application
**4**	Validation and testing	Performance testing using validation and test datasets; final adjustments	Finalized prototype ready for real-world testing

### 2.6 Validation

The application was validated using a holdout test set, and performance metrics were calculated, including accuracy, precision, recall, F1-score, sensitivity, and specificity. The validation process was informed by confusion matrices and classification reports to assess the model's ability to correctly classify the three categories of oral conditions.

### 2.7 Deployment pipeline and inference interface

The trained model was deployed as a web application branded as **Primary Referral and AI-based Screening for Assessing Oral Health (PRAYAAS)** using the Streamlit framework, enabling interactive access for users through a browser-based interface. In this setup, intraoral images are uploaded, resized to 224 × 224 pixels, and passed through the trained model in real time. The model returns a class prediction—normal/inflammatory, premalignant, or carcinoma—along with the corresponding confidence scores. The deployed model is hosted locally, with a model file size of approximately 95 MB and an average inference time of 0.9 s per image. This user-friendly web-based interface facilitates usability for non-technical users, particularly in research and pilot testing scenarios.

## 3 Results and findings

The AI-based application developed for the classification of Oral Potentially Malignant Disorders (OPMDs) demonstrated robust performance across training, validation, and testing phases, aligning with the Scrum-based methodology. The DenseNet201 model, selected as the best-performing architecture, showed significant accuracy and generalizability in classifying intraoral images into three categories: normal mucosa/inflammatory conditions, premalignant conditions, and oral carcinoma.

**Fig. 2:**
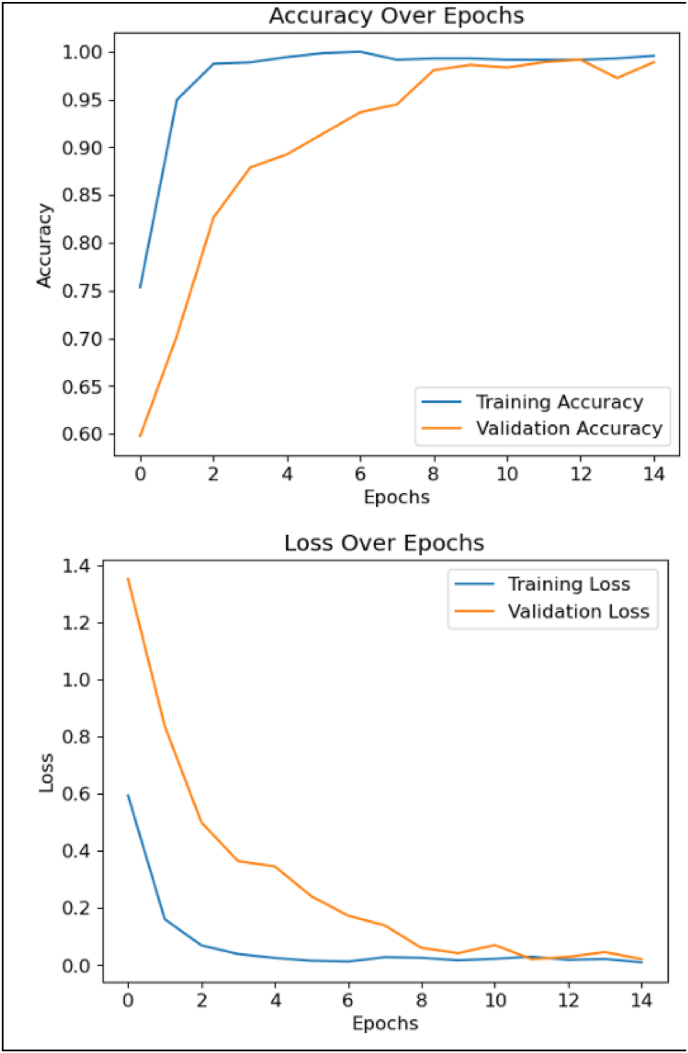
Training and validation performance over epochs.

The DenseNet201 model was trained on 70 % of the dataset (556 images), with 18 % (96) images) allocated for validation. During training, the model exhibited exceptional learning capabilities, maintaining a training accuracy of 99.5 % across all epochs and demonstrating minimal overfitting. Validation accuracy reached 98.9 % by the final epoch, underscoring the model's strong generalization to unseen validation data. The test accuracy, evaluated on a hold-out test set, was 94 %. These values are supported by the updated training and validation accuracy/loss curves presented in Fig. 2.

The model's robustness was further evaluated using an independent test dataset of 96 images. The overall test accuracy was 94 %, demonstrating reliable classification of OPMDs into the three categories: Category 1 (Normal Mucosa/Inflammatory Conditions), Category 2 (Premalignant Conditions), and Category 3 (Oral Carcinoma). Performance metrics for the test dataset are summarized in Table 2. For Category 1, the model achieved perfect precision, recall, and F1-scores of 1.00, reflecting no misclassifications. Similarly, for Category 2, the model achieved strong results, with a precision of 0.87, recall of 1.00, and an F1-score of 0.93, demonstrating its effectiveness in identifying premalignant lesions. However, for Category 3, while precision remained high at 1.00, recall dropped to 0.80, resulting in an F1-score of 0.89 due to minor misclassifications (as presented in Table 3).

**Table 3:** Performance metrics of DenseNet201 model on test dataset.

Category	Precision	Recall	F1-Score
Category 1: Normal Mucosa/Inflammatory	1.00	1.00	1.00
Category 2: Premalignant Conditions	0.87	1.00	0.93
Category 3: Oral Carcinoma	1.00	0.80	0.89

Key diagnostic metrics further assessed the model's performance. Sensitivity (true positive rate) was calculated as 0.8333 (95 % CI: 0.7000–0.9666), indicating that the model correctly identified 83.33 % of true positive cases, essential for early detection of OPMDs. Specificity (true negative rate) was 0.7143 (95 % CI: 0.6085–0.8201), showing the model's ability to minimize false positives and reduce unnecessary follow-ups. The positive predictive value (PPV) was 0.5556, reflecting the proportion of true positives among all positive predictions, while the negative predictive value (NPV) was 0.9091, demonstrating the model's reliability in ruling out OPMDs when results were negative. The F1-score, which balances precision and recall, was 0.67, reflecting the model's overall predictive ability.

**Fig. 3:**
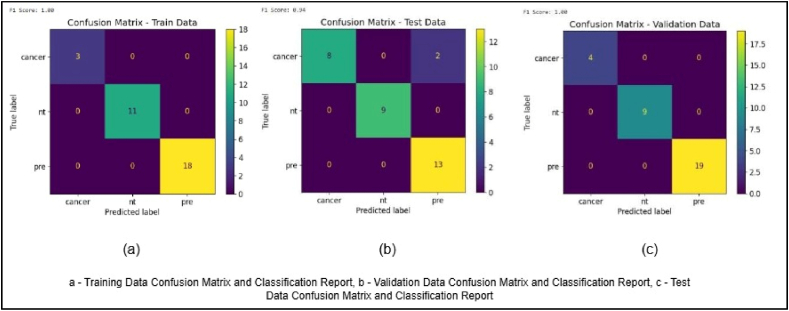
Confusion matrices for training, validation, and test subsets.

For visualization purposes, confusion matrices were generated from **balanced 32-image subsets (**Fig. 3) of the training, validation, and test datasets. These small subsets were perfectly classified (100 % precision/recall/F1), but the **overall validation accuracy for the full validation set was 87 %**, and the **independent test set accuracy was 94 %**. The 100 % values therefore reflect the small illustrative subsets and should not be interpreted as the full-dataset performance.

Each matrix presents the model's classification performance on a stratified subset of 32 images from the respective training, validation, and test datasets. These subsets were selected to ensure balanced representation of all three diagnostic categories—normal/inflammatory, premalignant, and carcinoma. The matrices serve as illustrative visualizations of class-wise prediction behaviour, while overall performance metrics are reported in Table 2.

Together, these metrics validate the PRAYAAS model as a valuable tool for early screening and risk stratification of OPMDs, especially in resource-limited settings. While the model performs reliably across all categories, further refinement may improve recall for challenging cases in the Cancer category, enhancing its clinical utility.

### 3.1 Workflow of the PRAYAAS application for OPMD detection

**Fig. 4:**
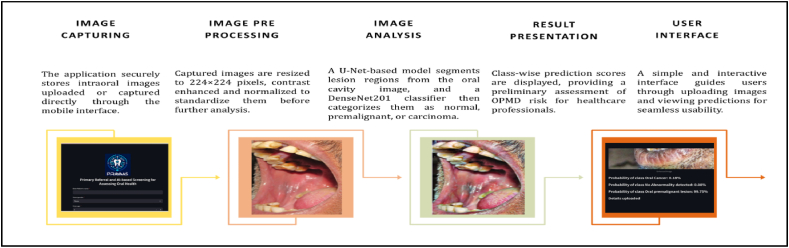
Workflow of the PRAYAAS application for OPMD detection and risk assessment.

The front end and user interface designed and developed for this algorithm was branded as “PRAYAAS” and acronym for “Primary Referral and AI-based Screening for Assessing Oral Health”.Fig. 4 presents the workflow of the PRAYAAS application, which is designed to detect Oral Potentially Malignant Disorders (OPMDs) through a systematic and automated process. The workflow begins with Image Capturing, where the application securely stores intraoral images uploaded by users or captured directly through the app. This serves as the foundation for subsequent analysis and ensures that the image data is readily accessible for processing.

In the Image Analysis stage, the application employs advanced machine learning algorithms to examine the uploaded images and identify potential signs of OPMDs. This step focuses on detecting abnormalities, such as unusual lesions or suspicious tissue patterns, that may indicate oral health risks. Following this, the workflow transitions to Image Segmentation, where the analysed images are categorized into one of three classes: normal variation of oral mucosa, premalignant conditions (e.g., leukoplakia or erythroplakia), or carcinoma (oral cancer). This classification is critical for accurate diagnosis and risk stratification.

Once the images are analysed and segmented, the Result Presentation stage displays a detailed assessment of OPMD risk. The application provides the probability of each category, allowing users to understand the likelihood of abnormalities and facilitating early intervention if needed. Finally, the User Interface ensures that all functionalities of the application are accessible through an interactive and user-friendly design. This intuitive interface enhances usability, making it suitable for healthcare professionals and non-specialist users alike. Overall, the PRAYAAS application workflow integrates secure image handling, robust machine learning analysis, and user-centric design to deliver an effective solution for early detection and risk assessment of OPMDs. This streamlined approach is particularly valuable for community screening programs and resource-limited settings.

The model achieved high accuracy across training, validation, and test datasets, with excellent precision and recall for normal mucosa and premalignant conditions. While minor misclassifications were observed in the carcinoma category during testing, the overall sensitivity, specificity, and F1-scores validate the model's reliability and diagnostic potential. Furthermore, the user-friendly workflow of the PRAYAAS application, from secure image capturing to detailed risk assessment, reinforces its suitability for real-world deployment.

## 4 Discussions

The dataset used in this study comprised 794 intraoral images, divided into 70 % for training (556 images), 18 % for validation (142 images), and 12 % for testing (96 images), aligning with standard practices for supervised deep learning workflows. Comparable studies, such as those by Dinesh et al.^14^ (360 images with 300 for training and 60 for testing) and Kouketsu et al.^15^ (1043 images, including 523 oral cancer images for training), employed similar or slightly larger image numbers. Likewise, Warin et al.^16^ used 980 images, a division of oral squamous cell carcinoma (OSCC), OPMD, and non-pathological cases, demonstrating a similar scale. However, some studies utilized significantly larger datasets, such as an ANN-based model trained on 11,981 pre-processed images or another model trained on 7000 CT images of early oral cancers. These large datasets often benefit from access to extensive medical databases and resources not always feasible in smaller studies.

Conversely, many studies relied on smaller datasets than this study, indicating the variability in resource availability across research. For instance, Baik et al.^17^ used only 62 images for training and Chen et al.^18^ used 161 images for training and 69 for testing, while Zhang et al.^17^ trained on just 38 images with 24 for testing. These smaller studies often faced limitations in dataset size, impacting model robustness and generalizability.

In comparison, the 794 images used in this study align well with the standards seen in the literature for AI-based OPMD classification.

The developed algorithm demonstrated a sensitivity of 0.8333 (95 % CI: 0.7000–0.9666), indicating a good but not optimal true positive rate compared to other AI models as presented in systematic review and meta-analysis by Uppal et al., 2024^19^. This is within the range of some AI models described in the literature but lower than the top-performing models that achieved 100 % sensitivity.^13^ The algorithm's specificity was 0.7143 (95 % CI: 0.6085–0.8201), suggesting a moderate ability to minimize false positives, which is lower compared to models with near 100 % specificity.^20^,^21^ The positive predictive value (PPV) was 0.5556, indicating that slightly over half of positive predictions were true positives, while the negative predictive value (NPV) was high at 0.9091. The F1-score, representing a balance between precision and recall, was 0.67. These results indicate an area for improvement in both specificity and overall balance of predictive power, and that the model should be used in conjunction with further clinical investigation to minimize false positive results and to improve patient outcomes.

The primary strength of this work lies in its development of an AI-based application tailored for early screening of OPMDs in resource-limited settings. The model demonstrated high accuracy in training and validation phases, with robust generalization to unseen data. The incorporation of a user-friendly interface enhances its accessibility for healthcare workers and non-specialist users, making it an effective tool for community-based screening programs. Additionally, the model's high NPV (0.9091) ensures that individuals classified as negative have a low likelihood of having undetected OPMDs, thereby reducing unnecessary interventions.

To address the identified limitations and enhance the model's clinical utility, several next steps are recommended. First, expanding the dataset to include more diverse and representative intraoral images from various demographic and geographic populations will improve the model's robustness and generalizability. Incorporating additional data augmentation techniques can also help simulate real-world variability and reduce the risk of overfitting. Second, further optimization of the model architecture, such as testing ensemble approaches or advanced architectures like transformers, may enhance sensitivity and specificity. Third, integration with real-time feedback mechanisms and continuous learning pipelines can allow the model to improve over time as it is deployed in clinical settings. Finally, conducting large-scale prospective studies to validate the model's performance in real-world settings will provide valuable insights into its efficacy and usability.

## 5 Conclusion

In conclusion, the PRAYAAS algorithm demonstrates significant potential as a diagnostic aid for OPMD detection, particularly in resource-limited settings. While its sensitivity, specificity, and PPV indicate areas for improvement, its strong NPV and F1-score highlight its reliability as a screening tool. With further refinement and validation, the application could serve as an impactful solution for early detection and risk stratification of OPMDs, contributing to improved oral cancer prevention efforts globally.

## Declaration of generative AI and AI-assisted technologies in the manuscript preparation process

During the preparation of this work the author(s) used ChatGPT4.0 in order to check the grammar. After using this tool, the authors reviewed and edited the content as needed and take full responsibility for the content of the published article.

## Ethical statement

This study utilized secondary anonymized data, which included clinical intraoral images that were previously de-identified and obtained from publicly available datasets and community screening settings. As the research did not involve direct interaction with human participants or access to identifiable private information, ethical approval was waived by the Institutional Review Board (IRB) at IIHMR Bangalore. This waiver aligns with institutional and international ethical guidelines for research involving secondary data analysis.

## Parents/gurdians cosents

Not Applicable.

## Source of funding

None.

## Declaration of competing interest

The authors declare the following financial interests/personal relationships which may be considered as potential competing interests:Akash Gajanan Prabhune reports administrative support, article publishing charges, and equipment, drugs, or supplies were provided by Institute of Health Management Research - Bangalore. If there are other authors, they declare that they have no known competing financial interests or personal relationships that could have appeared to influence the work reported in this paper.
